# Immediate Full‐Arch Implant Rehabilitation Through an Impacted Maxillary Canine: A 5‐Year Case Report

**DOI:** 10.1002/ccr3.73780

**Published:** 2026-10-21

**Authors:** Vladimir S. Todorovic, Miroslav Vasovic, Marija S. Milic

**Affiliations:** ^1^ School of Dental Medicine University of Belgrade Belgrade Serbia; ^2^ Oral Surgery Department, Faculty of Medical Sciences University of Kragujevac Kragujevac Serbia; ^3^ General and Oral Physiology Department, School of Dental Medicine University of Belgrade Belgrade Serbia

**Keywords:** dental implants, dentointegration, full‐arch rehabilitation, immediate loading, impacted canine, transdental implant placement

## Abstract

A deeply impacted tooth within a planned implant trajectory may complicate implant‐supported rehabilitation. Intentional transdental implant placement has been proposed as an alternative to extraction in selected patients. A 55‐year‐old woman with generalized advanced periodontitis, bruxism, and heavy tobacco use underwent bimaxillary fixed rehabilitation. Preoperative cone‐beam computed tomography (CBCT) analysis identified a deeply impacted maxillary left canine within the planned implant trajectory. Extraction was expected to create a substantial ridge defect that could compromise primary implant stability and necessitate augmentation and delayed implantation. Eleven implants were placed, including a 4.0 × 16 mm implant at position 25, inclined at 30° so that its apical portion traversed the impacted canine at position 23. All implants achieved insertion torque > 45 Ncm. Screw‐retained provisional full‐arch prostheses were delivered in full occlusal contact on postoperative Day 2 and definitive prostheses at 2 months. Analysis of the 2.5‐year CBCT identified crestal bone within 1 mm of the transdental implant shoulder. At 5 years, the implant remained asymptomatic, without clinical signs of peri‐implant inflammation. Qualitative panoramic assessment identified no pathology at the implant–tooth interface. Canine vitality remained undetermined, and no histological assessment was available. These findings do not establish dentointegration or quantify longitudinal bone‐level change.

## Key Clinical Message

An asymptomatic impacted maxillary canine does not necessarily preclude immediate fixed full‐arch rehabilitation. When extraction may compromise primary stability, intentional transdental placement may offer an alternative in carefully selected patients. Following immediate functional loading, the implant remained clinically stable, with no evident radiographic pathology at the 5‐year follow‐up.

## 1 Introduction

An impacted tooth within the planned implant trajectory may complicate implant placement. Extraction of a deeply impacted tooth may reduce alveolar bone volume, necessitate augmentation, increase surgical morbidity, and delay rehabilitation, potentially limiting immediate implant placement and loading [1]. Maxillary canines are among the most frequently impacted teeth after third molars, with a reported prevalence of 1%–2.5% [2]. Orthodontic traction is preferred in younger patients, although its predictability decreases with age [3].

Intentional implant placement through impacted teeth has been described as an alternative to extraction in selected patients since 2009. Although longer‐term outcomes have since been reported, the evidence remains limited to case reports and small series [1, 4, 5, 6, 7, 8, 9, 10]. Reported restorations include single crowns, fixed partial dentures, and fixed full‐arch prostheses [1, 11, 12]. A recent systematic review reported lower implant survival with preservation than with removal of the impacted structure, although this descriptive comparison does not establish the relative effectiveness of the two approaches [13]. Implant failures and infection have also been described [14, 15]. Table S1 summarizes selected published reports. To our knowledge, this is the first detailed case report of maxillary fixed full‐arch rehabilitation incorporating a transdental implant through an impacted canine under an immediate loading protocol. This report presents the rationale for treatment selection, surgical and prosthetic management, and 5‐year clinical and radiographic findings.

## 2 Case History and Examination

A 55‐year old woman was referred for bimaxillary implant‐supported rehabilitation. She presented with mandibular pain and swelling, impaired mastication, and discomfort associated with a maxillary porcelain‐fused‐to‐metal (PFM) bridge that had been in function for more than 15 years. Her medical and dental history included controlled hypertension, penicillin allergy, bruxism, and smoking approximately 50 cigarettes per day. Clinical and radiographic examination revealed generalized advanced periodontitis, a hopeless prognosis for the maxillary dentition, and periapical pathology affecting the mandibular dentition. Panoramic radiography and CBCT supported the poor prognosis of the dentition and identified a deeply impacted maxillary left canine within the planned implant trajectory (Figure 1).

**FIGURE 1:**
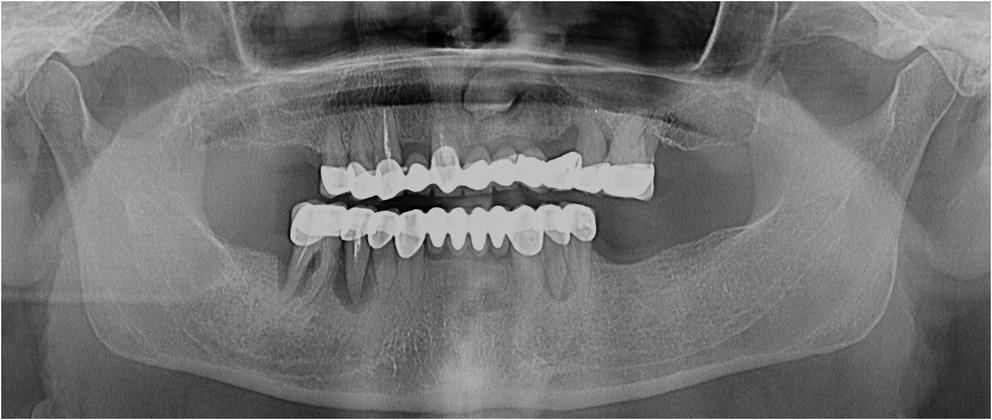
Preoperative panoramic radiograph demonstrating the remaining dentition, generalized periodontal bone loss, existing maxillary porcelain‐fused‐to‐metal (PFM) fixed partial denture (bridgework), and a deeply impacted maxillary left canine.

The fully impacted canine was asymptomatic, with bone and soft‐tissue coverage and no clinical signs of infection or radiographic evidence of periapical pathology. As pulp sensibility testing was not feasible and histological assessment was unavailable, its vitality remained undetermined. The decision to preserve the tooth was based on the absence of pathology rather than presumed vitality. Published selection criteria include a healthy, fully impacted tooth with at least 5 mm of coronal bone coverage [11], although the trajectory‐specific measurements in this case were not directly comparable with that criterion.

The patient preferred fixed rehabilitation within the shortest feasible timeframe, whereas conventional treatment was expected to require extraction, ridge reconstruction, and delayed implant placement. Table 1 summarizes the treatment timeline.

**TABLE 1:** Timeline of clinical management and follow‐up.

Time point	Clinical event
Baseline	Referral, clinical examination, and CBCT assessment
3 months before surgery	Periodontal hygiene phase and smoking‐reduction counseling
Day of surgery February 2021	Extraction of hopeless teeth and placement of 11 implants, including a transdental implant at position 25
Postoperative Day 2	Delivery of provisional screw‐retained full‐arch prostheses in full occlusal contact under an immediate loading protocol
2 months postoperatively	Delivery of definitive fixed prostheses
Every 6–8 months during follow‐up	Clinical follow‐up examinations
2.5 years postoperatively	CBCT assessment of peri‐implant bone levels and the implant–tooth interface
5 years postoperatively	Clinical examination and qualitative panoramic assessment. All implants remained in function.

Abbreviation: CBCT, cone‐beam computed tomography.

## 3 Methods

### 3.1 Treatment Planning and Decision‐Making

Preoperative CBCT analysis indicated that canine extraction could create a substantial ridge defect, compromise primary implant stability, and necessitate augmentation (Figure 2). This remained an imaging‐based inference because extraction was not performed. The alternative was intentional transdental placement to preserve native bone and achieve primary stability for immediate loading.

**FIGURE 2:**
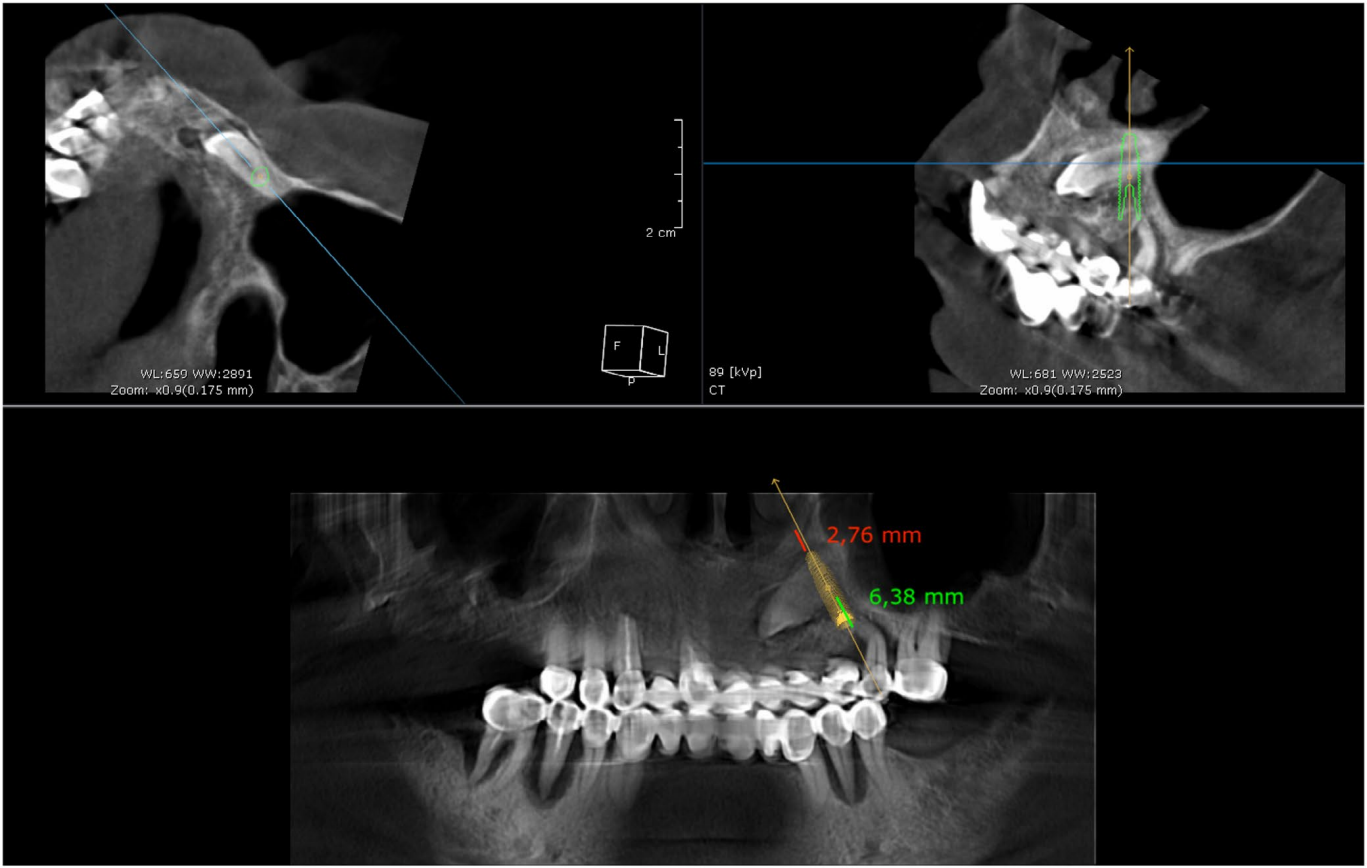
Preoperative cone‐beam computed tomography (CBCT) views illustrating the impacted maxillary left canine and the planned implant trajectory at position 25. Multiplanar views and a panoramic reconstruction illustrate the relationship of the planned implant to the canine root, alveolar crest, and floor of the nasal cavity. Along the planned trajectory, the distance from the alveolar crest to the first point of contact with the root was 6.38 mm, and the distance from the cranial boundary of the root to the floor of the nasal cavity was 2.76 mm. The implant outline and colored distance markers were added schematically for clarity. They are not direct exports of the software's measurement lines.

Both options, the unconventional nature of transdental placement, and risks of implant failure and infection were discussed before written informed consent was obtained. Given the limited long‐term evidence, the patient agreed to clinical and radiographic follow‐up, including three‐dimensional assessment of the transdental implant–tooth interface.

### 3.2 Quantitative CBCT Assessment

CBCT imaging was performed externally using a KaVo OP 3D Pro unit (PaloDEx Group Oy, Tuusula, Finland), with a 78 × 150 mm field of view and a 0.250 mm voxel size. Exposure parameters of 90 kV, 8 mA, and 4.5 s were supplied by the imaging center as its standard protocol rather than obtained from the archived study header.

Two examiners (Vladimir S. Todorovic, Miroslav Vasovic) performed measurements using OnDemand3D (Cybermed Inc., Seoul, Republic of Korea). The canine occupied a mid‐alveolar position, inclined at 40° to the occlusal plane. On multiplanar reconstructions aligned with the planned 30° implant trajectory at position 25, the crest‐to‐root contact distance was 6.38 mm, and the cranial root boundary was 2.76 mm from the nasal floor. The trajectory intersected the root canal (radicular pulp space); the osteotomy was prepared to accommodate an implant 16 mm in length and terminated within the root. Buccopalatal ridge width at position 25 was 6.10 mm. The extraction defect estimated from the radiographic outline was approximately 22.50 × 7.65 × 7.93 mm and was not an observed postoperative defect. Voxel sizes of 0.3–0.4 mm can provide diagnostically adequate images for implant planning, but voxel size alone does not establish measurement accuracy [16].

### 3.3 Terminology

Transdental implant placement denotes intentional insertion through the mineralized tissues of a retained impacted tooth, with transcanine specifying passage through a canine. Implant–tooth interface describes the observed anatomical relationship. Dentointegration and odontointegration refer to histologically documented integration between an implant surface and dental tissues [17, 18] and are not used to characterize this patient's outcome.

### 3.4 Preoperative Management

A 3‐month periodontal hygienic phase included scaling and root planing and oral‐hygiene reinforcement. Preoperatively, 0.2% chlorhexidine mouth rinse was prescribed twice daily. Chlorhexidine use continued during follow‐up. Smoking was reduced to < 10 cigarettes/day before surgery, although cessation was not achieved. For moderate dental anxiety, oral diazepam (5 mg) was prescribed for the evening before surgery and again 1 h preoperatively. Clindamycin (600 mg orally twice daily for 5 days) was initiated the evening before surgery.

### 3.5 Surgical Procedure

Surgery was performed in February 2021 under local anesthesia with 4% articaine and epinephrine 1:100,000 (Ubistesin Forte, 3 M Deutschland GmbH, 3 M ESPE, Seefeld, Germany). Full‐thickness flaps were elevated, and non‐restorable teeth were extracted, with alveoloplasty and debridement performed as indicated. Five Helix GM Acqua implants (Neodent, Curitiba, Brazil) were placed freehand at maxillary positions 15, 13, 11, 25, and 27. Implant angulations relative to the vertical were verified with an angulation gauge: 30° at positions 15, 25, and 27, 17° at 13, and axial at 11. GM Mini Conical abutments established a common path of insertion for the screw‐retained full‐arch prosthesis. At position 25, a 4.0 × 16 mm implant was tilted so that its apical portion traversed the impacted canine at position 23 (Figure 3). The manufacturer's drilling protocol was followed, with the osteotomy intersecting dental tissues and the radicular pulp space rather than merely engaging the tooth periphery. Continuous external saline irrigation was used throughout preparation [19]. Intraosseous temperature was not measured, precluding comparison of thermal effects with conventional osteotomy. Six mandibular implants with straight mini conical abutments supported a separate full‐arch prosthesis (Figure 4). Implant dimensions, angulations, and abutments are listed in Table 2.

**FIGURE 3:**
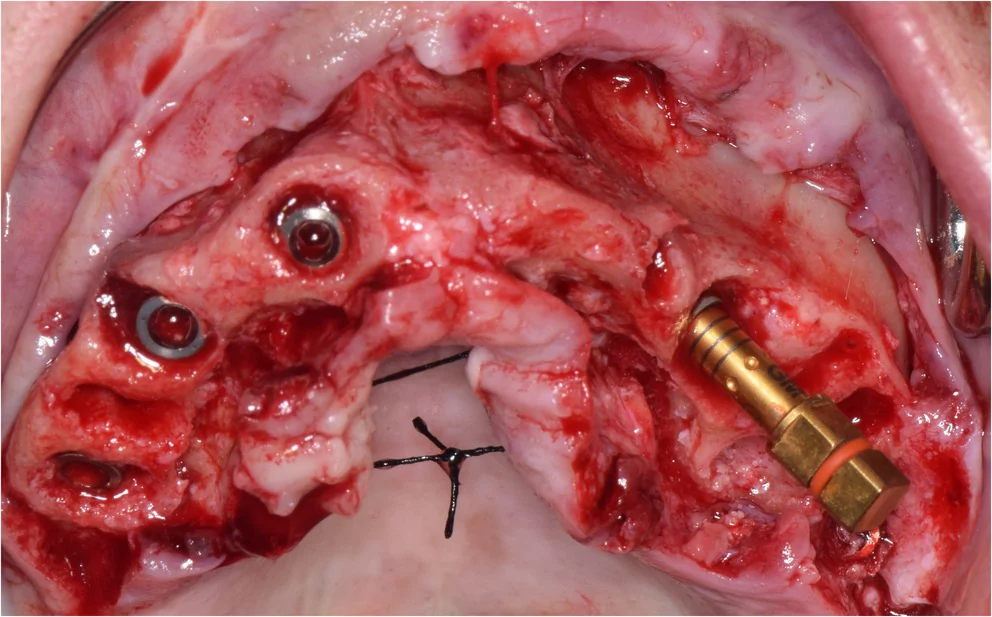
Intraoperative view following elevation of a full‐thickness mucoperiosteal flap, demonstrating placement of the 4.0 × 16 mm implant emerging at position 25, with its apical trajectory intentionally traversing the impacted maxillary left canine at position 23 during immediate full‐arch rehabilitation.

**FIGURE 4:**
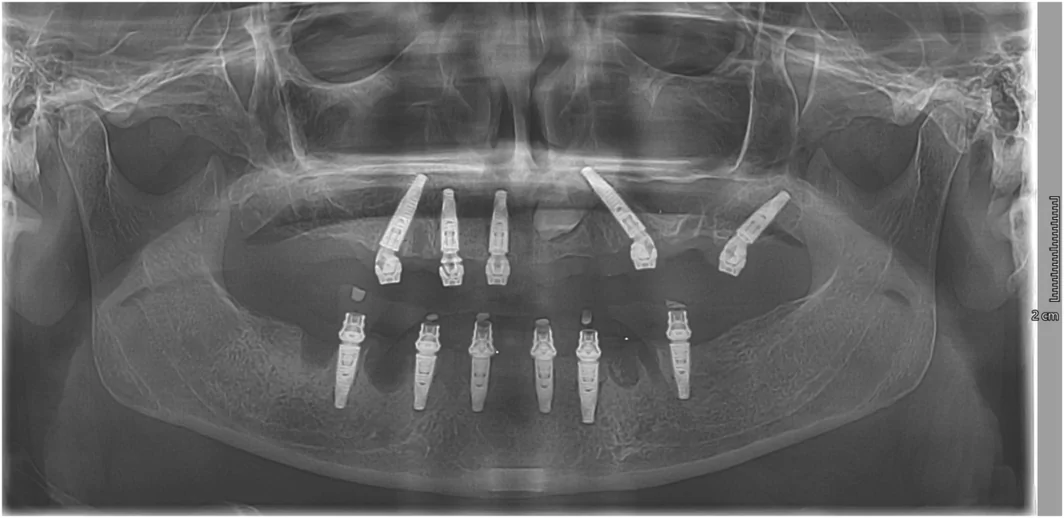
Immediate postoperative panoramic radiograph demonstrating full‐arch implant rehabilitation with five implants in the maxilla and six implants in the mandible, including the implant intentionally placed through the impacted maxillary left canine.

**TABLE 2:** Implant dimensions, angulations, and abutment specifications.

Arch	Position	Diameter (mm)	Length (mm)	Implant angulation^a^	Mini conical abutment
Maxilla	15	4.3	16	30°	30°
Maxilla	13	4.3	13	17°	17°
Maxilla	11	4.3	13	Axial	Straight
Maxilla	25 (transdental)	4.0	16	30°	30°
Maxilla	27	4.3	13	30°	30°
Mandible	36	4.3	11.5	Axial	Straight
Mandible	33	4.3	11.5	Axial	Straight
Mandible	32	3.5	11.5	Axial	Straight
Mandible	41	3.5	11.5	Axial	Straight
Mandible	43	4.3	11.5	Axial	Straight
Mandible	46	4.3	11.5	Axial	Straight

*Note:* All implants were Helix GM Acqua (Neodent, Curitiba, Brazil). Insertion torque exceeded 45 Ncm at every implant. Individual values were not recorded.

^a^ Angulation relative to the vertical, set and verified intraoperatively with an angulation gauge for angled implant placement and matched to the angulation of the mini conical abutment. All implants were placed freehand, without a surgical guide.

### 3.6 Criteria for Immediate Loading

Adequate primary stability is a prerequisite for an immediate loading protocol [20, 21]. All 11 implants, including the transdental implant, met the prespecified criterion with insertion torque exceeding 45 Ncm, although individual values were not recorded. Resonance frequency analysis was not performed, and implant stability quotient (ISQ) values were unavailable.

The five maxillary implants, including tilted posterior implants, were rigidly splinted with a screw‐retained provisional full‐arch prosthesis. Provisional prostheses were delivered on postoperative Day 2 in full occlusal contact under an immediate loading protocol. Given the patient's bruxism, a protective occlusal splint was provided for nocturnal use.

### 3.7 Follow‐Up Assessment

Follow‐up examinations every 6–8 months assessed implant mobility, pain or tenderness, peri‐implant inflammation, prosthetic integrity, peri‐implant bone levels, and the implant–tooth interface. Peri‐implant probing was performed routinely; however, probing depths, bleeding on probing, plaque scores, and bone‐sounding measurements were not systematically recorded and could not be reconstructed retrospectively. At 2.5 years, the implant–tooth interface was assessed three‐dimensionally using CBCT (Figure 5). Marginal bone levels were measured on the mesial and distal aspects from the implant shoulder to the first radiographically identifiable bone–implant contact on images reformatted along the implant axis. The implant portion within the canine root was measured along the implant axis. Final values were established by consensus between two examiners. At 5 years, repeat CBCT was not considered clinically indicated because the patient remained asymptomatic and clinically stable and the interface had already been assessed three‐dimensionally. Radiographic evaluation was therefore limited to panoramic imaging (Figure 6).

**FIGURE 5:**
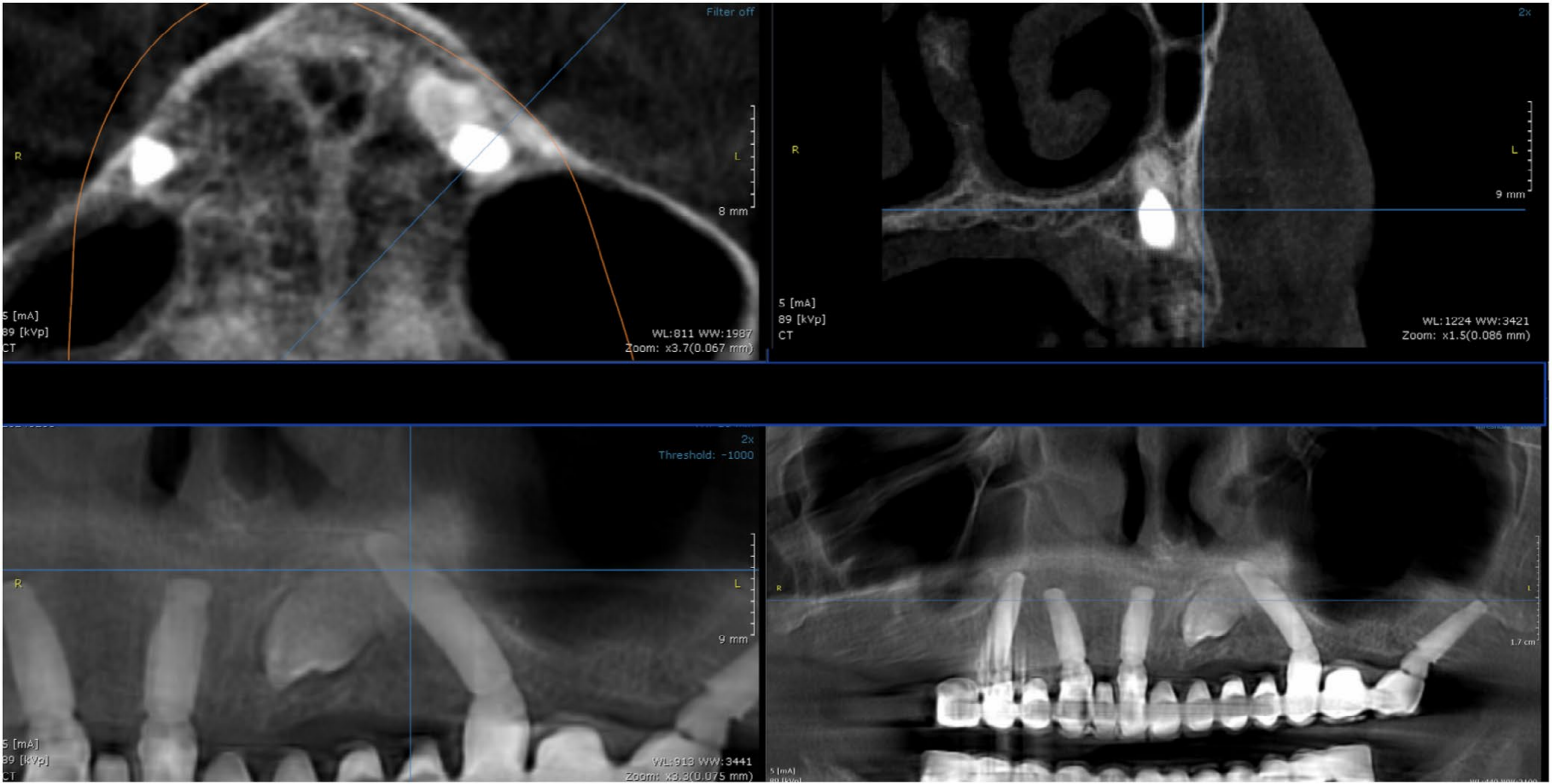
Cone‐beam computed tomography (CBCT) obtained 2.5 years after surgery demonstrating the implant intentionally placed through the impacted maxillary left canine. Multiplanar views demonstrate crestal bone levels and the surrounding alveolar bone architecture.

**FIGURE 6:**
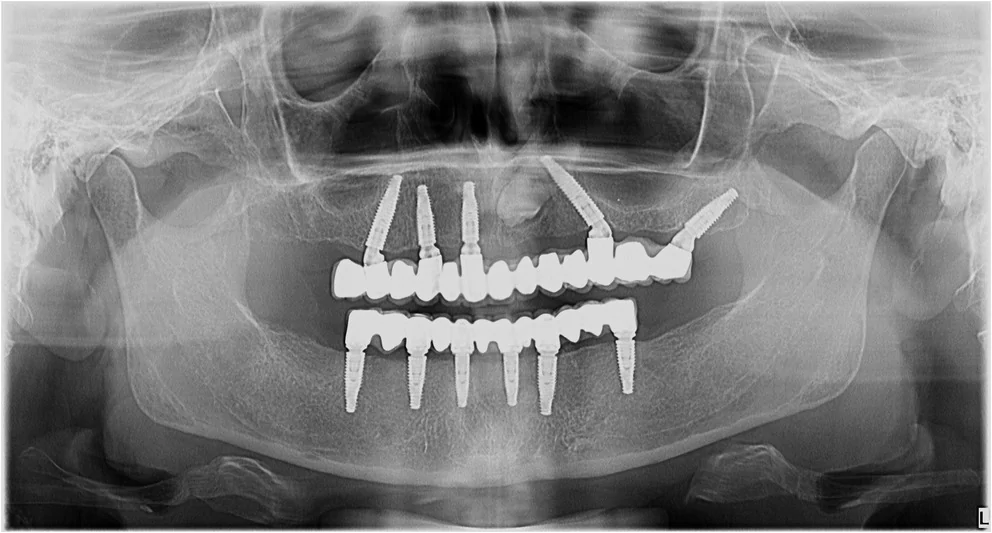
Panoramic radiograph obtained 5 years after surgery illustrating the full‐arch implant‐supported rehabilitation of both jaws, including the implant placed through the impacted maxillary left canine.

## 4 Results and Follow‐Up

Postoperative healing was uneventful, without unusual pain or early biological or mechanical complications. Definitive screw‐retained fixed prostheses replaced the provisionals at 2 months.

### 4.1 Tobacco Use During Follow‐Up

Smoking decreased from approximately 50 cigarettes/day at presentation to < 10/day at surgery and was reported as 10–20/day during the 5‐year follow‐up. Cessation was not achieved. Values were self‐reported as a range across follow‐up rather than at each visit and were not biochemically verified.

### 4.2 Imaging During Follow‐Up

At 2.5 years, the mesial and distal marginal bone levels at the transdental implant were 0.78 and 0.51 mm, respectively. Without a quantitative baseline, these single‐time‐point measurements could not establish annual bone loss or compliance with the < 0.2 mm criterion after the first year of function [22]. Reported radiographic precision (approximately 0.2 mm or worse) [23] does not establish CBCT measurement accuracy, and metal artifacts may obscure peri‐implant bone [24]. The mesial–distal difference was not interpreted, and crestal bone was described as lying within 1 mm of the implant shoulder.

CBCT analysis identified a 7.63 mm segment of the 16 mm implant traversing the impacted canine root, measured linearly along the implant axis. Dental tissues circumferentially enclosed part of this segment. The apex remained within the tooth, and the osteotomy intersected the radicular pulp space. The 5‐year panoramic radiograph was assessed qualitatively, without quantitative comparison with the 2.5‐year CBCT owing to differences in imaging modality and projection geometry.

### 4.3 Clinical Outcome at 5 Years

At 5 years, all 11 implants remained functional, without documented implant‐related biological or mechanical complications. The transdental implant remained immobile, painless, and without signs of peri‐implant inflammation. Analysis of the 2.5‐year CBCT and 5‐year panoramic images identified no peri‐implant radiolucency, apparent root resorption, or other pathological changes at the implant–tooth interface.

### 4.4 Patient Perspective

The patient's account was obtained at 5 years and was not used to assess clinical or radiographic outcomes.

“I am very satisfied with the treatment. My fixed teeth are much more comfortable than my previous prosthesis, and I can eat normally again. Although I was initially surprised by the idea of placing an implant through the impacted tooth, I never experienced any pain, discomfort, or other problems related to it. The treatment has significantly improved my quality of life, and if I had to make the same decision again, I would definitely choose the same treatment.”

## 5 Discussion

Intentional transdental implant placement enabled immediate full‐arch rehabilitation in this patient, whereas extraction was expected to compromise implant placement. Bone preservation relative to hypothetical extraction was not quantified, and neither dentointegration nor a contribution of dental tissues to primary stability was demonstrated.

### 5.1 Contribution to the Literature

Mandibular fixed full‐arch rehabilitation incorporating transdental implants has previously been reported with follow‐up of 8 years [1] and 6 years [11]. This case documents a 5‐year outcome following maxillary fixed full‐arch rehabilitation incorporating a transdental implant through an impacted canine, with full occlusal contact from postoperative Day 2. To our knowledge, this is the first detailed case report of this combination, although incomplete descriptions of prosthetic design and loading in some reports limit comparison [6, 10, 25]. Earlier maxillary rehabilitation involving an ankylosed canine root at the crest followed 6 months of submerged healing [1]. In the bilateral transcanine case reported by Smojver et al. [12], implant‐supported bridges were delivered after 3 months of healing, while interim restorations were supported by retained natural teeth. These differences in prosthetic support and loading time are relevant when comparing outcomes across reports.

Amato et al. [11] described non‐occluding provisional crowns, advised patients to avoid chewing for 8–10 weeks, and obtained definitive impressions at 6 months. The loading regimen of their mandibular full‐arch case was not separately specified. In this report, the transdental implant was rigidly splinted within a full‐arch prosthesis under immediate functional loading. Functional forces were not measured, precluding quantitative comparison of mechanical demands. Amato and Spedicato subsequently reported 14 maxillary implants in 10 patients, with follow‐up ranging from 3 to 11 years [10]. Their accessible abstract describes immediate loading but does not specify the prosthetic span or occlusal contacts, preventing direct comparison with the present full‐arch protocol.

Amato et al. recommended avoiding pulpal penetration [11], whereas a congress abstract reported pulp chamber penetration in 40/54 implants [6], and a geriatric case involved complete and partial tooth penetration [7]. In this case, a 7.63 mm segment of the implant traversed the canine root, with dental tissues circumferentially enclosing part of this segment. The apex remained within the tooth, and the osteotomy intersected the radicular pulp space. The contributions of bone and dental tissues to primary stability were not quantified, and these configurations cannot be assumed biomechanically equivalent. Insertion torque reflected resistance during placement through both bone and dental tissues. It did not distinguish the contribution of each tissue to initial fixation. Neither insertion torque nor the prosthetic outcome quantified the canine's mechanical contribution or load distribution among implants.

### 5.2 Reported Outcomes and Interface Biology

A single favorable outcome cannot establish safety or predictability. A systematic review reported success and survival rates of 85.18% and 92.59% with preservation of the impacted structure, versus 98.92% for both after removal [13]. The comparison was descriptive, and follow‐up duration differed between groups. A congress abstract reported six failures among 54 implants [6], with mobility, removal, failure, and infection reported elsewhere [4, 14, 15].

Histological findings of mineralized tissue formation and implant–dentin contact support biological plausibility [17, 18, 26, 27]. Evidence from retained‐root contact [28, 29, 30, 31] and socket‐shield procedures [32] does not establish predictability of transdental placement under functional loading. Without histology, dentointegration remains unconfirmed in this patient, and radiographic stability does not exclude microscopic changes. The histological evidence also arises from different settings, including animal implants contacting retained roots [17, 26, 27] and a human implant retrieved because of peri‐implant disease [18]. Tissue contact in these specimens cannot establish the long‐term behavior of a deliberately traversed, fully impacted canine. Differences in pulp involvement, surrounding periodontal tissues, and the distribution of bone and dental tissue limit direct extrapolation to this interface.

### 5.3 Osteotomy Preparation and Drill Wear

Drills used for transdental preparation exhibited greater visible wear than corresponding drills used exclusively in bone (Figure 7). Experimental studies associate drill wear with increased heat generation [19, 33], but neither wear nor temperature was quantified in this case.

**FIGURE 7:**
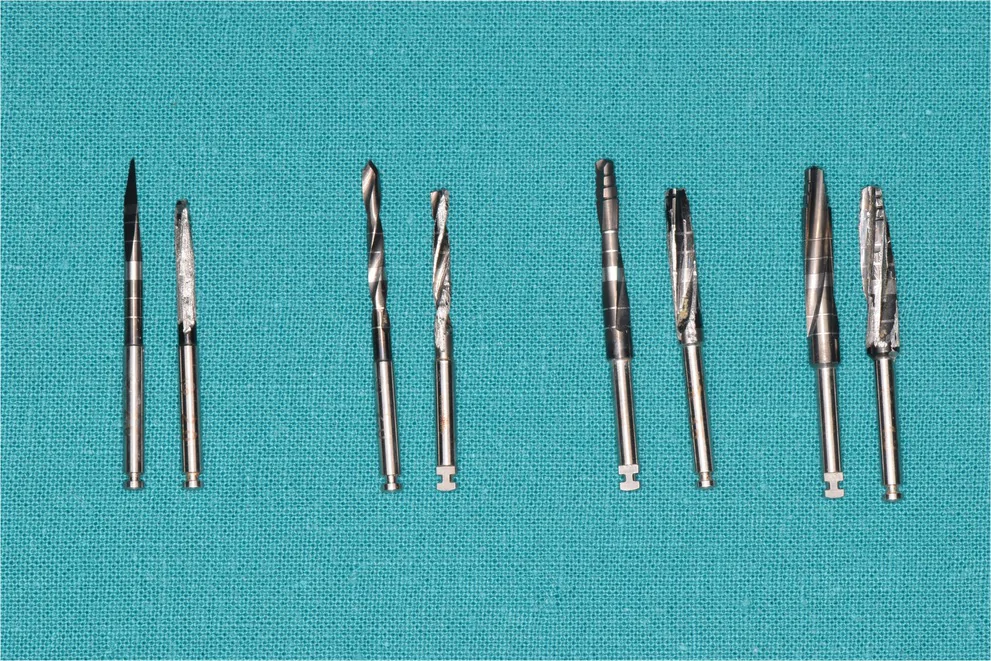
Comparison of drills used for transdental osteotomy (left of each pair) with corresponding drills used only for conventional osteotomy in alveolar bone (right). The drills used for transdental osteotomy exhibit visibly greater wear, including partial loss of the original machining marks and apparent shortening of the cutting portion. This observation is qualitative. No measurement of drill wear or intraosseous temperature was performed in this case.

Preparation was performed freehand, with angulation verified using a gauge. Adequate irrigation and assessment of drill condition remain reasonable precautions, although their thermal benefits were not evaluated. The absence of clinical complications does not exclude subclinical thermal injury. The photograph documents drill condition but does not isolate the effect of dental tissue from other determinants of wear. Drill design, prior use, drilling conditions, and irrigation can influence cutting performance and heat generation [19, 33]. The visual comparison therefore provides no quantitative basis for selecting a different drilling sequence or cooling protocol.

### 5.4 Case Selection

Selection criteria for transdental placement remain empirical [11]. A recent CBCT‐based classification includes this approach but lacks prospective validation [34], and the latest systematic review calls for further investigation [13]. The classification distinguishes extraction with implant placement, transcanine placement, and prosthetic alternatives when surgical risk is excessive [34]. It does not establish criteria for immediate functional loading through an impacted canine.

The proposed ≥ 5 mm coronal bone criterion [11] was not applied to the trajectory‐specific measurements because equivalence of the reference planes was not established. Despite penetration of the radicular pulp space, no symptoms were recorded over 5 years, but canine vitality and biological status remained undetermined.

This favorable outcome after bimaxillary rehabilitation does not clarify the risks associated with heavy smoking, advanced periodontitis, or bruxism. Immediate cross‐arch loading has been reported in heavy smokers [35]. A bimaxillary immediate‐loading series documented implant failures and provisional prosthesis fractures, with the latter occurring in patients with bruxism [36].

### 5.5 Limitations

Limitations include the single‐case design, lack of histology, canine vitality assessment and ISQ measurements, and incomplete soft‐tissue records despite routine probing. The 5‐year panoramic radiograph was not quantitatively comparable with the 2.5‐year CBCT. CBCT measurements may have been affected by metal artifacts [24] and lacked independently assessed accuracy and a quantitative baseline, precluding assessment of bone‐loss rate. Bone preservation relative to hypothetical extraction was unmeasured, smoking was self‐reported, and absence of complications does not exclude subsequent inflammatory, resorptive, or infectious events [14, 15]. Generalization to other anatomy or pathology is not justified.

## 6 Conclusion

In this patient, transdental implant placement through an asymptomatic impacted maxillary canine enabled fixed full‐arch rehabilitation under an immediate loading protocol, with favorable 5‐year clinical and radiographic outcomes. This approach warrants further investigation in selected cases where extraction may compromise implant placement. A single favorable outcome does not establish safety or predictability.

## Author Contributions

**Vladimir S. Todorovic:** conceptualization, methodology, investigation, data curation, visualization, writing – original draft, writing – review and editing, formal analysis. **Miroslav Vasovic:** writing – review and editing, investigation, formal analysis. **Marija S. Milic:** writing – review and editing.

## Funding

The authors have nothing to report.

## Ethics Statement

The treatment protocol complied with the ethical principles of the Declaration of Helsinki. The patient was informed of the unconventional nature of transdental implant placement and its alternatives before providing consent. This case report was prepared in accordance with the CARE guidelines [37].

## Consent

Written informed consent was obtained from the patient for publication of this case report and the accompanying clinical and radiographic images, including the quotation reported under Patient Perspective.

## Conflicts of Interest

The authors declare no conflicts of interest.

## Supporting information


**Table S1:** Selected reports relevant to prosthetic configuration and loading of transdental implants.

## Data Availability

The data that support the findings of this study are available on request from the corresponding author. The data are not publicly available due to privacy or ethical restrictions.
