# Supplementary material for: Immediate Full‐Arch Implant Rehabilitation Through an Impacted Maxillary Canine: A 5‐Year Case Report
Source: Clin Case Rep. 2026 Oct 21;14(10):e73780. doi: 10.1002/ccr3.73780 (PMC13686355; doi:10.1002/ccr3.73780)
Supplement: Supplementary file 1 [file CCR3-14-e73780-s001.docx]

**Supplementary Table S1**

Selected reports relevant to prosthetic configuration and loading of transdental implants.

| **Report** | **Arch and prosthetic configuration** | **Loading conditions** | **Follow-up** |
| --- | --- | --- | --- |
| Davarpanah et al. 2015 IV [1] | Maxillary single crown, mandibular fixed full-arch rehabilitation, and rehabilitation of an edentulous maxilla involving an ankylosed canine root at the crest. | Staged restoration after healing. Mandibular transdental implants healed for 3 months before prosthetic use. The maxillary full-arch case had 6 months of submerged healing. | 5–8 years, including 8 years for the mandibular full-arch case. Extended follow-up of cases previously reported in [4]. |
| Amato et al. 2019 [11] | Maxillary single-tooth restorations and a mandibular fixed full-arch case (case 3). Another partially edentulous case is illustrated. | Immediate provisional crowns were described as out of occlusion. The loading regimen of the mandibular full-arch case was not separately specified. | 5–7 years, including 6 years for case 3 |
| Smojver et al. 2021 [12] | Bilateral maxillary transcanine implants supporting screw-retained bridges within oral rehabilitation. Natural teeth were retained. | Implant-supported prosthodontic treatment after 3 months of healing. Interim bridges and crowns were supported by natural teeth. | 1 year after prosthodontic rehabilitation |
| Amato and Spedicato 2024 [10]† | Maxillary transdental implants. Prosthetic span is not specified in the accessible abstract. | Immediate loading is reported. Occlusal contacts and full-arch versus single-unit configuration cannot be established from the abstract alone. | 3–11 years (mean 7.2) |
| **Present case** | Maxillary fixed full-arch prosthesis incorporating an implant at site 25 through the impacted canine at site 23. A separate mandibular full-arch prosthesis was provided. | Provisional full-arch prostheses delivered in full occlusal contact on postoperative day 2 under an immediate loading protocol. | 5 years |

This focused, non-exhaustive comparison illustrates selected prosthetic and loading scenarios. It is not a systematic review and does not establish priority or comparative effectiveness.

† Details for Amato and Spedicato [10] are limited to the accessible published abstract. Unreported details are not treated as evidence that a treatment or loading condition was absent.

An additional full-arch transdental case is indexed as a congress abstract by Liu and Shi [25]. Its interim loading protocol could not be verified from the accessible record and is not used here to establish a difference in loading.

Reference numbers correspond to the reference list in the main manuscript.
